# The Preventive Mechanisms of Bioactive Food Compounds against Obesity-Induced Inflammation

**DOI:** 10.3390/antiox12061232

**Published:** 2023-06-07

**Authors:** Marta Pelczyńska, Małgorzata Moszak, Agnieszka Wesołek, Paweł Bogdański

**Affiliations:** 1 Chair and Department of Treatment of Obesity, Metabolic Disorders and Clinical Dietetics, Poznan University of Medical Sciences, 84 Szamarzewskiego Street, 60-569 Poznań, Poland; 2 Doctoral School, Poznan University of Medical Sciences, 10 Fredry Street, 61-701 Poznań, Poland

**Keywords:** inflammation, obesity, bioactive food compounds, polyphenols, omega-3 fatty acids, probiotics

## Abstract

Dietary patterns are promising strategies for preventing and treating obesity and its coexisting inflammatory processes. Bioactive food compounds have received considerable attention due to their actions against obesity-induced inflammation, with limited harmful side effects. They are perceived as food ingredients or dietary supplements other than those necessary to meet basic human nutritional needs and are responsible for positive changes in the state of health. These include polyphenols, unsaturated fatty acids, and probiotics. Although the exact mechanisms of bioactive food compounds’ action are still poorly understood, studies have indicated that they involve the modulation of the secretion of proinflammatory cytokines, adipokines, and hormones; regulate gene expression in adipose tissue; and modify the signaling pathways responsible for the inflammatory response. Targeting the consumption and/or supplementation of foods with anti-inflammatory potential may represent a new approach to obesity-induced inflammation treatment. Nevertheless, more studies are needed to evaluate strategies for bioactive food compound intake, especially times and doses. Moreover, worldwide education about the advantages of bioactive food compound consumption is warranted to limit the consequences of unhealthy dietary patterns. This work presents a review and synthesis of recent data on the preventive mechanisms of bioactive food compounds in the context of obesity-induced inflammation.

## 1. Introduction

Obesity is a disease characterized by the excessive amount or distribution of adipose tissue (AT) in the human body [1]. In the International Classification of Diseases, ICD-11, obesity is 5B8 [2]. Obesity affects over one billion people worldwide. The majority of people with obesity are adults, reaching 650 million people in the world. Around 340 million adolescents and 39 million children are obese [3]. Excessive body weight is associated with over 2.8 million deaths annually [4]. Obesity increases the risk of hospitalization and generates high healthcare costs in most countries. As an epidemic of the 21st century, it is a challenge to public health [5].

An anthropometric indicator, body mass index (BMI), is used to diagnose obesity. It is a simple, cheap, quick, and non-invasive diagnostic tool. BMI is the quotient of body weight expressed in kilograms and the square of height in meters (kg/m^2^). A score above 30 kg/m^2^ makes it possible to diagnose obesity. Waist circumference is also used for the diagnosis of the discussed disease. A waist circumference of ≥94 cm in men or ≥80 cm in women is diagnostic of abdominal obesity [6]. This disease can also be diagnosed by determining the percentage of AT using the electrical bioimpedance method. Growth charts are also used in children and adolescents [7].

The most common cause of obesity is an imbalance between the energy consumed from food and the energy expended. This is due to incorrect eating habits, the avoidance of physical activity, and sleep deficiency. External factors such as family and school also play a significant role [8]. Childhood obesity is also diagnosed as a secondary complication of other diseases. These include Prader–Willi, Bardet–Biedl, and Alström syndromes [9]. Obesity is related to many aspects of the patient’s life. These may include eating disorders [10], differences in social behavior and living conditions, social inequalities [11], stress [12], and psychiatric disorders [13]. Its pathogenesis is multifactorial [14].

Excessive levels of AT increase the risk of numerous disorders. The complications of obesity include diabetes, coronary artery disease, hypertension, non-alcoholic fatty liver disease, stroke, cataract, and fertility problems [6]. Obesity can also cause lipid metabolism disorders, such as low levels of high-density lipoprotein (HDL-C) and an increase in triglyceride concentration (TG) [15]. It also increases the risk of hospitalization and death from COVID-19 [16] and the risk of cancer, including colorectal cancer [17]. Individuals with obesity also face stigma related to the disease [18]. As a consequence, obesity shortens life expectancy [19].

Adipose tissue performs many significant physiological functions in the human body. An excessive amount of AT results in pathological conditions in many organs and systems. AT is also a significant endocrine organ. It shows morphological and physiological diversity [20]. Obesity generates chronic inflammation caused by excess body AT [21]. Visceral adipose tissue (VAT) and ectopic accumulations of adipocytes are particularly metabolically active [22]. Adipocytes synthesize numerous peptides called adipokines and cytokines, such as leptin, adiponectin, tumor necrosis factor-α (TNF-α), interleukin 6 (IL-6), plasminogen activator inhibitor 1 (PAI-1), monocyte chemotactic protein 1 (MCP-1), resistin, adipsin, apelin, and visfatin [23,24]. An excessive number of adipocytes causes disorders in the concentrations of these molecules. Consequently, it predisposes affected individuals to the development of metabolic disorders and generates low-grade inflammation [25].

Dysfunctions of adipokines and AT play the most significant role in inducing chronic inflammation in the human body. Nutrition is another factor responsible for both its intensification and inhibition. It depends on applied dietary patterns. Incorrect eating habits, including foods that are highly processed, rich in simple sugars and saturated fatty acids, and low in vitamins, microelements, and dietary fiber, may intensify this process [26]. On the other hand, greater consumption of whole grains, fruits, vegetables, legumes, nuts, and olive oil significantly reduces the concentration of inflammation biomarkers [27]. These contributions are part of an anti-inflammatory diet. Its role is to reduce inflammatory processes by inhibiting cell pathways, attenuating the secretion of proinflammatory molecules, or increasing the secretion of anti-inflammatory agents. In addition, the large number and variety of bioactive food compounds with high biological activity are responsible for the anti-inflammatory potential of this diet. These compounds are food ingredients or dietary supplements other than those necessary to meet basic human nutritional needs and are responsible for positive changes in human health [28]. These include, i.a. polyphenols, omega-3 fatty acids, and probiotics [29].

This review discusses the importance of the preventive mechanisms of bioactive food compounds in the context of obesity-induced inflammation. The literature review was conducted between February and April 2023 using the National Library of Medicine browser (Medline, PubMed).

## 2. Oxidative Stress in Obesity

Excessive amounts of AT in the human body generate low-grade chronic inflammation. The accumulation of adipocytes impairs the functionality of several metabolic pathways. This takes place both in the AT itself and in other organs. In an obese state, the level of circulating free lipids is increased. Metabolic changes may initiate a state of insulin resistance (IR). However, likewise, IR can increase inflammation and the accumulation of adipocytes. Reactive oxygen species (ROS) and reactive nitrogen species (RNS) are among the most potent contributors to inflammation in the body. They are produced in the mitochondria, endoplasmic reticulum, lysosomes, peroxisomes, and cytosolic enzymes [30,31]. Complexes I and III of the electron transport chain in mitochondria generate ROS. Excess ROS levels in the mitochondria may lead to their dysfunction and consequently initiate IR, type 2 diabetes, non-alcoholic fatty liver disease, myocarditis, and heart failure [31,32]. Glutathione deficiency in adipocytes leads to the formation of a redox circuit by pyruvate dehydrogenase (PDH) and nicotinamide nucleotide transhydrogenase (NNT). This may consequently produce H_2_O_2_ [33]. Additionally, the very structure of adipose tissue generates ROS. As previously mentioned, proinflammatory factors are generated in the case of its excess. These include cytokines and adipokines such as TNF-α, IL-6, angiotensin II, PAI-1, leptin, visfatin, and resistin. These proinflammatory adipokines modulate, in turn, IR. They affect the insulin signaling pathway directly or indirectly by stimulating inflammatory pathways. Obesity also reduces the concentration of adipokines, which have a positive effect on increasing insulin sensitivity. Thus, adiponectin, IL-10, and omentin concentrations decrease [34]. Low-grade inflammation in AT is strongly correlated with significant changes in the profile of secreted adipokines. This is a hallmark of obesity’s pathophysiology.

Interleukin 6 is one of the human body’s most essential and multidirectional cytokines. Monocytes and macrophages secrete it. Its main functions include stimulating inflammatory processes. The increase in the IL-6 level induced by obesity decreases insulin sensitivity in the liver, muscles, pancreas, and white adipose tissue (WAT). The primary source of IL-6 in obesity is the increase in proinflammatory type 1 macrophages (M1) [35]. In people with average body weight and normal adipose tissue contents, the number of type II macrophages (M2) with anti-inflammatory activity is dominant. As a result of M1 and M2 level disorders, in addition to increased IL-6 secretion, inducible nitric oxide synthase (iNOS) and NO secretion are activated. Moreover, the secretion of other proinflammatory cytokines, such as IL-12 and IL-13, is observed. As a result of the decrease in the concentration of M2, the level of the anti-inflammatory cytokine IL-10 decreases. M2 releases IL-10. Disturbances in M1 and M2 levels affect insulin sensitivity in obesity. Stimulated M1 also causes an increase in TNF-α secretion. This promotes the formation of NO. TNF-α also increases IR, reduces the insulin receptor substrate 1 (IRS-1) concentration and peroxisome proliferator-activated receptor gamma (PPAR-γ) expression, and activates nuclear factor kappa-B (NF-κB) [36].

The nitric oxide radical (NO•) seems to be a well-known free radical, serving important physiological functions in the human body. It is synthesized from arginine produced by citrulline oxidation by three main isoforms of nitric oxide synthase (NOS). The first one, epithelial NOS (eNOS), is related to vascular regulation and vasodilation; the second one, neuronal NOS (nNOS), is responsible for intracellular signaling; and the third one, inducible NOS, is involved in the body’s response to different endotoxin or cytokine signals [37]. Thus, NO, when maintained at low or moderate levels, is crucial in supporting human health. It is an important agent modulating cell-to-cell responses and regulating blood flow thrombosis and neural activity [38]. On the other hand, under proinflammatory conditions, signaling cascades triggered by TLR engagement enhance the expression of iNOS and promote the production of NO [39]. The concomitant generation of O_2_ may react with excess NO to form another reactive molecule, peroxynitrite (ONOO−), and other toxic RNS. Therefore, the activation of TLRs may result in both oxidative and nitroxidative stress [40].

TNF-α secretion in obesity is increased by leptin and resistin levels. Leptin is a hormone affecting the energy balance and the feeling of starvation and satiety. It regulates appetite and metabolism by inhibiting neuropeptide Y (NPY) synthesis and release in the arcuate nucleus (ARC). Increasing the concentration of TNF-α increases the expression of leptin in AT. Higher levels of leptin increase the concentrations of IL-6, IL-12, and C-reactive protein (CRP). The leptin concentration depends on body weight, nutrition, and the amount of AT. Increasing the amount of AT in obesity causes a chronic increase in leptin concentration, affecting the progression of chronic inflammation [41]. Resistin, in turn, is an adipokine that affects glucose–insulin metabolism. Its secretion induces the synthesis of TNF-α, (IL)-1β, IL-6, IL-8, and IL-12 through the NF-κB-mediated pathway. The binding of resistin to adenylate cyclase (CAP-1) increases the expression of NF-κB, cAMP, and protein kinase A (PKA). This has the effect of inducing a proinflammatory response [42].

Plasminogen activator inhibitor 1 is an inhibitor of the fibrinolytic system. Its increased concentration is a predictor of myocardial infarction. It is produced in ectopic adipose tissue by macrophages. PAI-1 concentrations in the blood depend on the AT distribution in the body. The level of PAI-1 positively correlates with the marker of oxidative stress (OS). Increasing ROS production in AT impairs PAI-1 secretion in obesity. The increased expression of PAI-1 by ROS may be prevented by a dominant negative inhibitor of NF-κB [43]. Lowering the PAI-1 concentration seems to be promising for lowering IR in patients with obesity by improving insulin sensitivity in adipose tissue.

Chronic OS also affects the formation of WAT, appetite regulation, increased preadipocyte proliferation, adipocyte differentiation, and the size of mature adipocytes [44]. H_2_O_2_ levels in WAT are controlled by catalase, glutathione peroxidase (GPX), and peroxiredoxins (Prdxs). Peroxiredoxin 3 (Prdx3) intercepts H_2_O_2_ in WAT and reduces OS. In obesity, the level of Prdx3 is reduced. This increases the oxidative imbalance in WAT [45].

To sum up, visceral adipose tissue accumulation leads to abdominal obesity. VAT exerts the most substantial proinflammatory effect. Its amount is strongly correlated with IR [46]. Therefore, in obesity, the AT distribution in the body is also essential in addition to its excess [46]. The increased amount of AT induces the synthesis of the above-mentioned proinflammatory cytokines and adipokines, promoting increased ROS and nitrogen production by macrophages and monocytes. Changing nutrition, following a diet with antioxidant potential, and reducing body weight seem to be promising strategies for attenuating the secretion of proinflammatory factors in obesity [47]. Moreover, it seems that bioactive food compound intake can improve and accelerate the obesity treatment process and reduce inflammation.

## 3. Antioxidant Potential of Bioactive Food Compounds in Obesity Management

As mentioned above, increased AT causes increased proinflammatory cytokine expression and ROS generation. This condition is aggravated by dietary patterns rich in simple sugars and saturated fatty acids. The opposite effect is exerted by a diet high in bioactive food ingredients [48].

Bioactive food compounds include many ingredients that have diverse effects on the human body. However, these compounds show the strongest antioxidant activity. Their importance is crucial in reducing obesity-induced inflammation. Consuming products containing bioactive food components may reduce inflammation and OS in the human body [49,50,51]. An appropriate composition of anti-inflammatory agents in the diet may represent a new approach to obesity treatment [52]. These compounds are mainly found naturally in plant products. These include polyphenols, unsaturated fatty acids, lactic acid bacteria, vitamins, dietary fiber, certain trace elements, and oligopeptides [27]. These compounds suppress the NF-κB/MAP kinase pathway and maintain or slightly increase the level of M2 macrophages, which induces the production of anti-inflammatory cytokines. They also affect the regulation of proinflammatory adipokines [44], increase the level of NAD in the cell, inhibit lipid synthesis, and increase energy expenditure and thermogenesis [53]. Anti-inflammatory compounds are also available as dietary supplements [54].

Polyphenols are a diverse group of bioactive food ingredients. These are organic compounds of plant origin. Berries, colorful vegetables, green tea, cocoa, and nuts are the primary sources of these compounds. Both animal and clinical studies have demonstrated the health benefits of food-derived polyphenols in obesity. Polyphenols may improve the functioning of the cardiovascular system, have anti-inflammatory effects, and normalize the lipid profile and blood pressure value. Resveratrol (RSVL) has an antioxidant effect and supports the immune system, inhibits lipogenesis, reduces inflammation in obesity, and increases energy expenditure [55]. Catechins present in green tea attenuate the proliferation of 3T3-L1 adipocytes by reducing the levels of phosphorylated ERK1/2, cdk2, and cyclin D1 proteins. They also inhibit cell growth in Go/G1 and induce apoptosis in mature adipocytes. Phenolic compounds present in blueberries reduce the expression of TNF-α and IL-10 genes in macrophages, which decreases inflammatory processes [56].

The following nutritional compounds with antioxidant capacity are dietary fats. Fatty acids are divided into saturated and unsaturated (mono- and polyunsaturated) depending on the presence and number of unsaturated bonds. Omega-3 fatty acids have the strongest anti-inflammatory effect. It has been shown that supplementation with n-3 fatty acids reduces the concentration of interleukins IL-1 and IL-6 and prostaglandin (PG) and cytokine levels. Consuming more n-3 fatty acids than in a standard diet affects the anti-inflammatory cytokine response [57].

In recent years, special attention has been paid to the human microbiome and its influence on metabolic health and the inflammatory response. Lactic acid bacteria are capable of obligately fermenting carbohydrates to form lactic acid. Lactobacillus bacterial strains have numerous beneficial health aspects. In the diet, they are found in fermented foods. The lactic acid produced by these bacteria has an antioxidant effect. This leads to the reduction of ROS. It also affects the production of antioxidants, superoxide dismutase (SOD), catalase (CAT), and glutathione (GSH) [58]. Lactobacillus bacterial strains also affect the lowering of IL-1 and IL-6 [59].

Bioactive food compounds also include vitamins A [60], C [61], and D [62,63] and dietary fiber. Dietary fiber is an element of plant products not digested by human digestive enzymes. These include, in the most significant amounts, cellulose, hemicelluloses, pectins, gums, lignins, and cutins. They significantly affect the intestinal microflora. Dietary fiber has an antioxidant effect mainly due to the high contents of phenolic compounds. Their amount depends on the species and the part of the plant. Dietary fiber reduces the production of IL-6 and IL-8 in humans as well as the production of endothelial proinflammatory cytokines by inhibiting NF-κB and proteasome activity [64]. Of the oligopeptides showing antioxidant activity, glutathione has been the most studied. This is a cellular non-enzymatic antioxidant. It is a cofactor for glutathione peroxidase, glutathione S-transferase, and glyoxalase [65]. A good dietary source of glutathione is spinach, which was first shown in 1978 [66].

Dietary foods and/or supplements contain many numerous antioxidant compounds. Each of them represents a different antioxidant effect on humans. Thus, this review describes the antioxidant potential of selected polyphenols, omega-3 fatty acids, and probiotics against obesity-induced inflammation.

## 4. Polyphenols

**Figure 1:**
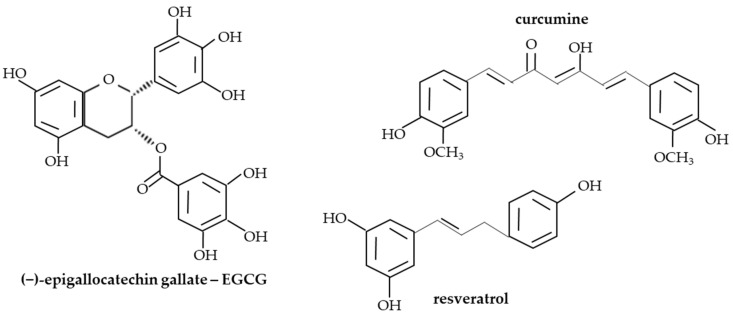
The structures of selected polyphenols.

**Figure 2:**
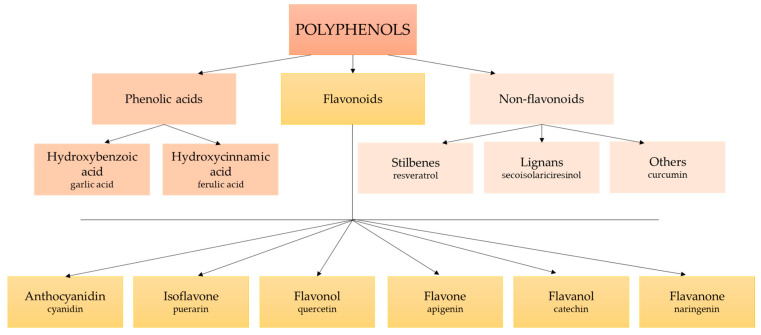
The classification of polyphenols.

Polyphenols or phenolic compounds are plant metabolites arising from the polyketide acetate/malonate pathway, the shikimate/phenylpropanoid pathway, or both [67]. In nature, around 8000 molecules have been identified as polyphenols [68]. This term refers to compounds with one or more hydroxyl substituents bound to aromatic rings. Their structure is very varied, ranging from simple molecules (such as phenolic acids) to complex polymers with high molecular weight (such as tannins) (Figure 1) [69,70]. There are many different classifications of polyphenols, although the main categorization distinguishes between groups of flavonoids and non-flavonoids, including stilbenes, lignans, phenolic acids, and others (Figure 2) [50,71]. Polyphenols are the most abundant phytochemicals in the plant kingdom, with a prominent presence in fruits and vegetables. Their bioavailability depends on many factors, such as the kind and amount of the food, the intestinal condition, interactions with other food products and/or medicines, and the pharmacokinetic profile [53,72]. The presence of one or more aromatic rings in the molecule and a different number of hydroxyl groups determine the antioxidant activity of polyphenols. Thus, the chemical structure determines the rate of absorption and the nature of its metabolites circulating in the plasma. Consequently, the biological properties of polyphenols differ from one to another, and their absorption through the gut barrier is correlated with an increase in antioxidant capacity [73].

It has been shown that polyphenols have antioxidant, anti-inflammatory, immunomodulatory [74], anti-cancerogenic [75,76], and anti-obesity [29,77] properties. Although their exact mechanisms of action are still not fully understood, emerging data suggest their positive influence on human health. The main effect of the action of phenolic compounds is their ability to increase the expression and activity of antioxidant enzymes and inhibit the production of free radicals [31,78]. Moreover, they can modulate cell functions in obesity, especially the number and size of adipocytes (inhibition of adipogenesis), control lipid metabolism and fatty acid oxidation, and inhibit lipid accumulation [78,79]. Due to the large number of different polyphenols compounds, in this review, we describe resveratrol, curcumin (CUR), and catechins in the context of the attenuation of obesity-induced inflammation.

### 4.1. Resveratrol

Resveratrol, 3,4′,5-trihydroxystilbene, is a non-flavonoid polyphenol compound found in grapes (mainly grape skin), red wine, peanuts, and some berries (blueberries and cranberries). Plants synthesize RSVL in response to severe conditions, that is, UV irradiation, fungal infections, or injuries [80]. There are two isometric forms of RSVL: trans, primarily in grape skin and grape juice, and cis in red wine. Although both forms have high biological activity and similar antioxidant properties, the main object of interest is trans-RSVL due to the unstable form of the cis configuration [81]. Importantly, although RSVL is well absorbed by the human digestive system, its bioavailability is relatively low because of its rapid metabolism and excretion [82]. The concentration of RSVL (both resveratrol and its metabolites) 30 min after its oral administration (25 mg/70 kg men) varies between 416 and 471 μg/L and depends on the source of RSVL (vegetable juice, grape juice, red wine) [83]. The content of RSVL in red wine (2–12.6 mg/L) is higher than in grapes (0.24–1.25 mg/cup/160 g) or grape juice (1.14–8.69 mg/L), which results from the fermentation of grape skin used to produce wine. Nevertheless, it has been suggested that eating grapes and/or drinking grape juice is sufficient for RSVL intake without consuming alcohol [82,84,85].

RSVL shows antioxidant and anti-inflammatory activity and has beneficial effects in preventing and treating metabolic disorders such as obesity [86]. Studies conducted on cell cultures (especially AT culture models—3T3-L1 adipocytes) showed that RSVL might inhibit adipogenesis by reducing the expression of PPAR-γ through ubiquitin-dependent proteasome degradation [87,88]. It also prevents lipid (triglyceride) accumulation due to an increase in the liver’s expression of sirtuin 1 (SIRT 1), a molecule regulating energy metabolism and mitochondrial homeostasis in cells [89]. RSVL decreases lipogenesis in adipocytes through the downregulation of lipogenic genes, such as lipoprotein lipase (LPL), sterol regulatory element-binding protein-1c (SREBP1c), fatty acid synthase (FAS), and stearoyl-CoA desaturase-1 (SCD1) [88,90]. Activated by resveratrol, AMP-activated protein kinase (AMPK) phosphorylates and blocks acetyl-CoA carboxylase, which results in a decrease in the synthesis of malonyl-CoA, a stimulator of lipogenesis [91]. Cell culture studies also showed that RSVL increases lipolytic activity in human [92] and rat [93] adipocytes via an increase in cyclic adenosine monophosphate (cAMP) levels. It has been pointed out that this effect was potentiated when RSVL administration was combined with genistein [94]. Thus, it seems that RSVL may enhance fatty acid β-oxidation, mitochondrial biogenesis, and their activity [95].

The anti-obesity properties of RSVL may also result from its anti-inflammatory response in AT. It was found that RSVL (0.1–10 μM) pretreatment reduced the secretion of TNF-α and IL-6 from 3T3-L1 adipocytes, as well as suppressed inflammatory-related proteins such as NF-κB and extracellular receptor-activated kinase (ERK; Table 1) [96]. In addition, this bioactive compound also inhibits proinflammatory molecules stimulated by IL-1β expression; these include IL-6, IL-8, PAI-1, MCP-1, and other adipokines (such as leptin) in 3T3-L1 cells [97,98].

Animal studies also indicated the potential positive effect of RSVL administration on reducing AT inflammation. These studies were based mainly on diet-induced obese animal models. The results from different studies indicate that RSVL administration not only attenuates obesity-induced chronic inflammation (i.a. by attenuating the expression of proinflammatory molecules, such as IL-6, TNF-α, or interferon (IFN-γ and IFN-β)) [99,100] and inhibits OS (i.a. by decreasing malondialdehyde (MDA) and glutathione disulfide (GSSG) levels) [101] but also enhances the antioxidant capacity (i.a. by increasing the activity of liver SOD or catalase) [99,101]. Kim et al. confirmed the anti-inflammatory effect of RSVL in vitro in an experimental AT mouse model. In this study, RSVL attenuated high-fat-diet-induced (HFD) inflammation in mouse WAT by inhibiting the levels of proinflammatory cytokines (such as TNF-α and IL-6) and their upstream signaling molecules (such as NF-κB; Table 1) [100]. The analyzed compound may also reduce macrophage infiltration into AT in Zucker rats [92], as well as lead to a decrease in the proinflammatory M1 phenotype (CD11cþ) together with an increase in M2 polarity (CD206þ) in the WAT of sleep apnea mice [102]. In research conducted on primates, high-fat, high-sugar (HFHS) diet-fed adult rhesus monkeys showed that RSVL supplementation decreases adipocyte size and the mRNA levels of IL-6, TNF-α, and IL-1β; increases SIRT1 expression; inhibits NF-κB activation; and improves insulin sensitivity in the VAT of animals (Table 1) [103].

Clinical studies indicated that RSVL intake might positively affect obesity-induced inflammation. A systematic review and meta-analysis of randomized controlled trials (RCT = 24) conducted by Tabrizi et al. showed that RSVL supplementation significantly decreases hs-CRP (standardized mean difference (SMD), −0.55; 95% CI, −0.84, −0.26; *p* < 0.001; I2: 84.0) and TNF-α levels (SMD, −0.68; 95% CI, −1.08, −0.28; *p* = 0.001; I2: 81.3) among patients with metabolic syndrome, with no changes in Il-6 and SOD concentrations [104]. Similar results were provided by another meta-analysis of seventeen studies (n = 736), which showed significant reductions in the levels of TNF-α (weighted mean difference (WMD), −0.44; 95% CI, −0.71 to −0.164; *p* = 0.002; Q statistic = 21.60; I2 = 49.1%; *p* = 0.02) and hs-CRP (WMD, −0.27; 95% CI, −0.5 to −0.02; *p* = 0.033; Q statistic = 26.95; I2 = 51.8%; *p* = 0.013) after RSVL supplementation [105]. More observational studies evaluated the anti-obesity effect of RSVL not only due to its anti-inflammatory properties but also due to other pathways. In a randomized, double-blind crossover study, the authors observed that 150 mg of RSVL per day induced metabolic changes (i.a. increased energy expenditure and decreased AT lipolysis and plasma fatty acids) in obese humans, mimicking the effects of calorie restriction (Table 1) [106]. Other authors reported that 30 days of RSVL treatment (150 mg/day) significantly decreased adipocyte size and improved AT function in obese men [107].

**Figure 3:**
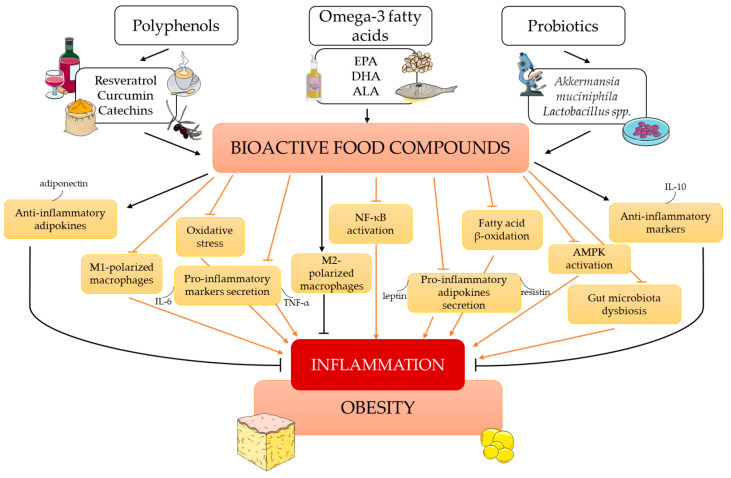
The influence of bioactive food compounds on obesity-induced inflammation. Abbreviations: ALA, α-linolenic acid; AMPK, AMP-activated protein kinase; DHA, docosahexaenoic acid; EPA, eicosapentaenoic acid; IL-6, interleukin 6; IL-10, interleukin 10; NF-κB, nuclear factor kappa B; TNF-α, tumor necrosis factor-α. This figure was made using the Servier Medical Art collection (http://smart.servier.com/) (accessed on 24 April 2023).

To sum up, the potential anti-obesity mechanisms of resveratrol include the inhibition of preadipocyte differentiation, a reduction in adipocyte proliferation, and the induction of adipocyte apoptosis. Moreover, RSVL decreases lipogenesis, enhances lipolysis and fatty acid β-oxidation, and limits AT inflammation (Figure 3) [81,85,108].

**Table 1:** Polyphenols and their effects on inflammation in obesity (results from selected in vitro, animal, and human studies).

Bioactive Compound	Experimental Model	Results	References
**Resveratrol**	RAW264.7 cells and 3T3-L1 cells treated with RSVL (0.1, 1, 10 μM) for 1 h	RAW264.7 cells: ↓ LPS-stimulated IL-6 and TNF-α synthesis 3T3-L1 cells: ↓ proinflammatory factor (TNF-α, IL-6) production and changes in adipokine mRNA expression (upregulation for adiponectin and downregulation for resistin) ↓ NF-κB activation and ERK1/2 phosphorylation ↓ Phosphorylation of IRS-1 and AKT ↑ Insulin sensitivity in adipocytes	Kang et al. [96]
	In vitro model of human AT treated with RSVL (10, 30, 100 μM) for 48 h	↓ IL-6, IL-8, and MCP-1 levels in a concentration-dependent manner in adipocytes under inflammatory conditions ↓ NF-κB activity	Zagotta et al. [97]
	Male C57BL/6J mice (9 weeks old) on an HFDThree groups (n = 10), i.e., SD, HFD, and HFD + RSVL (0.4% RSVL), treated for 10 weeks	↓ BW gain, ↓ visceral fat-pad weight, and ↓ TG, FFA, TC, glucose, TNF-α, and MCP1 concentrations ↓ Galanin-mediated signaling molecules (GalR1/2, PKCd, Cyc-D, E2F1, p-ERK) ↓ Key adipogenic gene expression (PPAR-g2, C/EBPα, SREBP-1c, FAS, LPL, aP2, leptin) in the epididymal AT ↓ Proinflammatory cytokines (TNF-α, IL-6, IFN-α, IFN-β) and their signaling molecules (NF-κB, TLR2/4, MyD88, Tirap, TRIF, TRAF6, IRF5, p-IRF3)	Kim et al. [100]
	Male C57BL/6 mice (6 weeks old) Mice were randomly divided into four groups (n = 10) and treated for 18 weeks as follows: SD, HFD (41.26% of calories from fat), HFD-RSVL/L (200 mg/kg/day), HFD-RSVL/H (400 mg/kg/day)	RSVL (400 mg/kg/day) ↓ Insulin resistance, ↓ TC, TG, LDL concentrations, ↑ HDL level, ↑ expression of pAkt, GLUT4, and IRS-1 in WAT, and ↓ serum proinflammatory cytokine levels (MCP-1, TNF-a, and IL-6), macrophage infiltration, and CCR2 expression in WAT	Ding et al. [109]
	High-fat, high-sugar diet-fed adult (7–13 years old) rhesus monkeys Monkeys were quasi-randomized into one of three groups and treated for 2 years: HF-HS diet + RSVL (n = 10), HF-HS diet + placebo (n = 10), and SD (n = 4) RSVL supplementation in doses of 80 mg and 480 mg/day for the first and second years, respectively	↓ Adipocyte size and mRNA levels of IL-6, TNF-α, and IL-1β, ↑ SIRT1 expression, ↓ NF-κB activation, and ↑ insulin sensitivity in VAT of HF-HS animals	Jimenez-Gomez [103]
	Diabetic patients (n = 94) were randomly assigned to RSVL (n = 45) or placebo (n = 46) groups supplementing once daily with 200 mg of RSVL or cellulose capsules for 24 weeks, respectively A randomized, double-blinded, placebo-controlled parallel group trial	↓ Plasma glucose, ↓ insulin, ↓ HOMA-IR, ↓ MDA, ↓ hs-CRP, ↓ TNF-α, and ↓ IL-6 RSVL supplementation regulated diabetes-associated miRNA levels (more than two-fold downregulation of miRNA-34a, miRNA-375, miRNA-21, and miRNA-192 and upregulation of miRNA-126 and miRNA-132 expression)	Mahjabeen et al. [110]
	Healthy, obese men (n = 11) supplementing with 150 mg of RSVL per day Randomized double-blind crossover study (30 days)	Activation of AMPK, ↑ SIRT1 and PGC-1α protein levels, ↑ citrate synthase activity in muscles, ↑ intramyocellular lipid levels, ↓ intrahepatic lipid content, ↓ circulating glucose and insulin levels, ↓ HOMA-IR value, ↓ TG, ↓ ALAT, and ↓ SBP and inflammation markers (↓ IL-6, IL-8, TNF-α) In the postprandial state: ↓ lipolysis, plasma fatty acid, and glycerol level in AT	Timmers et al. [106]
Curcumin	Human monocytic THP-1 cells pretreated with CUR for 1 h and subsequently induced with PMA for 48 h Incubation of cells with CUR (in a dose of 0–100 μg) for 24-48 h	↓ NLRP3 inflammasome expression, ↓ caspase-1 activation, ↓ IL-1β secretion, ↓ TLR4 expression, and ↓ NF-κB activation	Kong et al. [111]
	TNF-α-stimulated 3T3-L1 adipocytes treated with 2–20 μM of curcumin (or RVSL) for 62 h	↓ NF-κB activation, ↓ TNF-α, IL-1β, IL-6, and COX-2 gene expression, and ↓ IL-6 secretion	Gonzales et al. [112]
	HFD-induced obese (n = 5) and leptin-deficient ob/ob male C57BL/6J mice (n = 5) Standard diet (4% fat) ± curcumin 3% by weight HFD (35% fat) ± curcumin 3% by weight for 6 weeks	↑ Foxo1 and adiponectin expression, ↓ infiltration of macrophages, ↑ circulating adiponectin levels, and ↓ MCP-1 in WAT ↓ TNF-α and MCP-1 expression and NF-κB activity in liver	Weisberg et al. [113]
	Male C57BL/6J mice (n = 12/group) LFD (10% kcal from fat), HFD (45% kcal from fat), and HFD + curcumin (4 g/kg diet) added 2 days/week for 28 weeks	↓ Macrophage infiltration ↓ NF-κB expression and JNK signaling pathway activation in AT	Shao et al. [114]
	Obese individuals (males and females, n = 30) receiving 1g of CUR per day Randomized, double-blind, crossover	↓ IL-1β, ↓ IL-4, and ↓ VEGF ↔ Other proinflammatory cytokine levels (e.g., IL-1, IL-6, TNF-α)	Ganjali et al. [115]
	Overweight/obese subjects with MetS (males and females, n = 117) taking 1 g/day of CUR (n = 59) or placebo (n = 58) for 8 weeks Randomized, double-blind, placebo-controlled trial	↓ TNF-α, ↓ IL-6, ↓ TGF-β, and ↓ MCP-1	Panahi et al. [116]
Catechins	Palmitate-induced 3T3-L1 adipocytes incubated with epicatechin (EC) (0.1, 1 μM) for 24 h VAT from HFD-fed mice on a diet supplemented with 20 mg of EC per kg of body weight for 15 weeks	↓ TNF-α, ↓ IL-6, ↓ MCP-1, ↑ adiponectin, ↓ F4/80, and ↓ NF-κB	Bettaieb et al. [117]
	Adipocytes co-cultured with LPS-induced macrophages incubated with 50 μM of EC for 1 h WAT from HFD-fed C57BL/6J mice supplemented with 20 mg of EC per kg of body weight for 12 weeks	↓ TNF-α, ↓ IL-6, ↓ CCL19, ↓ Rantes, ↓ Ip-10, ↓ Saa3, ↓ Lbp, and ↓ Socs3 ↓ TNF-α, ↓ IL-6, ↓ MCP-1, and ↓ Saa3	Sano et al. [118]
	TNF-α- and GC-(4->8)-GCG-induced 3T3-L1 adipocytes (10, 20 μg/mL) for 24 h Serum and WAT from HFD-fed male C57BL/6 mice supplemented with GC-(4->8)-GCG in a dose of 40 or 80 mg/kg/day for 8 weeks	↓ IL-6, ↓ COX-2, ↓ MCP-1, ↓ TNF-α, ↓ F4/80, ↓ CD11b, ↓ NF-κB, ↓ JAK, ↓ STAT3, and ↓ MAPKs	Peng et al. [119]
	Female SD rats (3 months old) on an HFD (n = 12/group) Groups included LFD and HFD or HFD + 0.5% w/v GTCs for 8 months (4 months with GTCs)	↓ BW and %FM and ↑%FFM ↓ IGF-I, ↓ leptin, and ↓ proinflammatory cytokines (IL-1α, IL-2, IL-4, IL-10, GM-CSF, IFN-γ, TNF-α) ↑ GPX protein expression in liver	Shen et al. [120]
	Obese, hypertensive subjects (n = 56) taking GTCs (379 mg GTCs/d) or placebo for 3 months Double-blinded, placebo-controlled trial	↓ Inflammation and oxidative stress (↓ TNF-α, ↓ CRP, ↑ TAS) ↔ BMI, waist circumference, creatinine, and glucose ↓ SBP and DBP ↓ TCH, LDL-C, and TG and ↑ HDL-C ↓ Serum insulin and ↓ HOMA-IR	Bogdański et al. [121]
	Obese patients with metabolic syndrome (n = 35) consuming green tea (928 mg total catechins in 4 cups/d) or GTCs (870 mg total catechins in 2 capsules/d) or placebo (4 cups water/d) for 8 weeks Randomized controlled trial	↔ Inflammatory markers (adiponectin, hs-CRP, IL-6, IL-1β, sVCAM-1, sICAM-1, leptin, and leptin/adiponectin ratio) ↔ Waist circumference, SBP, DBP, TG, HDL, and glucose ↓ Plasma serum amyloid alpha	Basu et al. [122]

Abbreviations: ACC, acetyl-coenzyme A carboxylase; ACO, acetyl-CoA oxidase; AT, adipose tissue; AKT, serine/threonine protein kinase; ALAT, alanine aminotransferase; AMPK, AMP-activated protein kinase; Ap-1, activator protein 1; aP2, adipocyte protein 2; ATF-2, activating transcription factor 2; ATGL, adipose tissue triglyceride lipase; BMI, body mass index; BW, body weight; CCL19, chemokine (C-C motif) ligand 19; C/EBP, CCAAT/enhancer-binding protein; COX2, cyclooxygenase 2; CPT-1, carnitine palmitoyltransferase-1; CREBBP, CREB-binding protein; CUR, curcumin; Cyc-D, cyclin D; DBP, diastolic blood pressure; E2F1, E2F transcription factor 1; EC, epicatechin; eNOS, endothelial nitric oxide synthase; ERK1/2, extracellular signal-regulated protein kinases 1 and 2; F4/80, macrophage marker; FABP4, fatty acid-binding protein 4; FASN (FAS), fatty acid synthase; FFA, free fatty acid; FFM, free fat mass; FM, fat mass; FOXO1, forkhead box protein O1; GalR, galanin receptor; GLUT4, glucose transporter type 4; GM-CSF, granulocyte-macrophage colony-stimulating factor; GPX, glutathione peroxidase; GSSG, glutathione disulfide; GTCs, green tea catechins; HDL-C, high-density lipoprotein cholesterol; HFD, high-fat diet; HF-HS diet, high-fat, high-sugar diet; HNF-4 α, hepatocyte nuclear factor receptor-4α; HOMA-IR, homeostasis model assessment: insulin resistance; hs-CRP, high-sensitivity C-reactive protein; IFN, interferon; IL, interleukin; Ip-10, interferon-gamma-inducible protein 10; IRF5, interferon regulatory factor 5; JAK, Janus kinase; JNK, Jun NH2-terminal kinase; IGF-I, insulin-like growth factor-I; IRF, interferon regulatory factor; IRS-1, insulin receptor substrate-1; KLF2, Krüppel-like factor 2; Lbp, lipopolysaccharide binding protein; LDL-C, low-density lipoprotein cholesterol; LFD, low-fat diet; LPL, lipoprotein lipase; LPS, lipopolysaccharide; MAPK, mitogen-activated protein kinase; MCE, mitotic clonal expansion; MCP-1, monocyte chemoattractant proteinMyD88-1; MDA, malondialdehyde; ME, malic enzyme; MetS, metabolic syndrome; Mfn2, mitofusin 2; MMP, matrix metalloproteinase; MyD88, myeloid differentiation primary response gene 88; NEFA, non-esterified fatty acid; NF-κB, nuclear factor kappa B; NO, nitric oxide; NOS, nitric oxide synthase; OLETF, Otsuka Long Evans Tokushima Fatty; PAI-1, plasminogen activator inhibitor-1; pAkt, phosphorylated protein kinase B; p-ERK, phosphorylated extracellular signal-related kinase; PGC-1α, peroxisome proliferator-activated receptor-coactivator 1a; PI3K, phosphatidylinositide 3-kinase; PKCd, protein kinase C delta; PMA, phorbol 12-myristate 13-acetate; PMBC, peripheral blood mononuclear cell; PPAR, peroxisome proliferator-activated receptor; Rantes, normal T cell expressed and secreted; RSVL, resveratrol; Saa-3, serum amyloid A3; SBP, systolic blood pressure; SCD1, stearoyl-CoA desaturase-1; SD, standard diet; sICAM-1: soluble intercellular adhesion molecule-1; SIRT1, sirtuin (silent mating-type information regulation 2 homolog) 1; Socs3, suppressor for cytokine signaling 3; SOD, superoxide dismutase; SREBP-1, sterol regulatory element-binding protein-1; STAT3, signal transducer and activator of transcription 3; TAS, total antioxidant status; TC, total cholesterol; TGF-β, transforming growth factor β; TBARS, thiobarbituric acid-reactive substances; TFAM, mitochondrial transcription factor A; TG, triglyceride; Tirap, toll-interleukin 1 receptor (TIR) domain-containing adaptor protein; TLR, toll-like receptor; TNF-α, tumor necrosis factor alpha; TRAF6, TNF receptor-associated factor 6; TRIF, TIR-domain-containing a; WAT, white adipose tissue; VAT, viscera adipose tissue; VEGF, vascular endothelial growth factor; ↑, increase; ↓, decrease; ↔, no change.

### 4.2. Curcumin

Curcumin (CUR) is a bioactive non-flavonoid compound extracted from turmeric (*Curcuma longa*). The latter is a spice widely consumed in India and other Asian countries. The nutritional value of 100 g of turmeric represents around 354 kcal, 8 g of protein, 19 g of fats (with no cholesterol), 65 g of carbohydrates (including 21 g of fiber and 3 g of sugar), and minerals such as sodium (38 mg) and potassium (about 2.5 g) [123]. The beneficial health effects of turmeric are associated with curcuminoids, a group of chemically related low-molecular-weight polyphenols containing around 77% CUR, 17% demethoxycurcumin, and 3% bidemethoxycurcumin [124]. Moreover, it was indicated that turmeric contains more than 100 bioactive compounds [125]. Curcumin, the most studied turmeric component, is characterized by good tolerance (even at dosages of up to 12 g/day with no side effects) by humans [126]. It is estimated that the average intake of turmeric is 2 g/day (among adult Indians), which corresponds to 200 mg of CUR [127]. Curcumin is weakly absorbed in the intestines, although other natural phytochemicals, such as piperine, increase its bioavailability [128].

Curcumin has well-documented anti-inflammatory, antioxidant, anti-obesity, anti-angiogenic, and anti-carcinogenic activities [129,130]. CUR plays an important role in regulating enzymes, cytokines, kinases, receptors, growth factors, transcription factors, and metastatic and apoptotic molecules in different phases of the development of many diseases, such as obesity [131].

Ejaz et al. examined CUR’s in vitro and in vivo effects on 3T3-L1 adipocytes and HF mice on a diet supplemented with a 500 mg CUR/kg diet for 12 weeks. In vitro, CUR (5–20 μmol/L) suppressed 3T3-L1 differentiation by suppressing the phosphorylation of mitogen-activated protein kinases (mitogen-activated protein kinases (MAPKs), ERK, c-Jun N-terminal kinases, and p38 MAPKs), caused apoptosis, and inhibited adipokine-induced angiogenesis. For HF mice, supplementation with CUR did not affect food intake, reduced body weight gain and adiposity, and decreased the expression of vascular endothelial growth factor (VEGF) and its receptor. Moreover, CUR decreased the serum cholesterol level and inhibited the expression of PPAR-γ and CCAAT/enhancer-binding protein, which are critical transcription factors involved in adipogenesis and lipogenesis [132]. In another study conducted with the use of 3T3-L1-derived adipocytes (with or without TNF-α stimulation), CUR and RSVL treatment reduced NF-κB activation, as well as caused a reduction in IL-1β, IL-6, TNF-α, and cyclooxygenase 2 (COX-2) gene expression (inhibitory concentration—IC50 = 2 muM). Moreover, the study showed a reduction in secreted IL-6 and PGE2 (IC50 = 20 muM), key mediators of the inflammatory response (Table 1) [112].

Weisberg et al. found that CUR ameliorated diabetes by improving glucose and insulin tolerance and hemoglobin A1c (HbA1c) values in HF-diet-induced obese (n = 5) and leptin-deficient *ob*/*ob* male C57BL/6J mice (n = 5). CUR treatment (3% by weight of admixture of CUR) significantly reduced macrophage infiltration in WAT, increased adiponectin production, and reduced NF-κB activity, as well as the concentration of markers of hepatic inflammation (i.a. MCP-1; Table 1). The authors also indicated that mice from CUR groups (vs. controls) consumed significantly more daily food, representing a lower body weight and body adipose tissue [113]. Other authors indicated that CUR significantly decreased body weight/fat gain, glucose disposal, and IR development in HFD mice. In addition, CUR blocks the effects of HFD on macrophage infiltration and the inflammatory and oxidative pathways in AT and attenuates lipogenic gene expression in the liver (Table 1) [114].

The effect of CUR on obesity-induced inflammation has been widely evaluated in clinical trials. In an RCT conducted by Ganjali et al. on obese individuals (n = 30), it was shown that CUR consumption in a dose of 1 g for 4 weeks resulted in a significant reduction in IL-1β (*p* = 0.042), IL-4 (*p* = 0.008), and VEGF (*p* = 0.01) concentrations, with no effect on other proinflammatory cytokines’ levels (e.g., IL-1, IL-6, TNF-α; Table 1) [115]. Another double-blind RCT involved 84 overweight/obese patients with non-alcoholic fatty liver disease (NAFLD), and it was observed that CUR supplementation (two 40 mg capsules/day after meals for 3 months) caused a reduction in proinflammatory markers, such as TNFα, hs-CRP, and IL-6 (*p* < 0.05), as well as significant positive changes in many biochemical (i.a. lipid profile and glucose indices) and anthropometric parameters (waist circumference) [133]. Similar conclusions were also reached in other clinical studies [116,134] and meta-analyses [135,136]. Ferguson et al., after analyzing 32 trials (n = 2038), showed reduced hs-CRP (WMD, −1.55 mg/L; 95% CI, −1.81 to −1.30), IL-6 (WMD, −1.69 pg/mL, 95% CI, −2.56 to −0.82), TNF-α (WMD, −3.13 pg/mL; 95% CI, −4.62 to −1.64), IL-8 (WMD, −0.54 pg/mL; 95% CI, −0.82 to −0.28), and MCP-1 levels (WMD, −2.48 pg/mL; 95% CI, −3.96 to −1.00), as well as an increased IL-10 concentration (WMD, 0.49 pg/mL; 95% CI, 0.10 to 0.88), with no effect on the ICAM-1 level after CUR supplementation [135]. Additionally, Gorabi et al., in a meta-analysis of 32 RCTs, reported a significant decrease in the serum levels of IL-1 (WMD, −2.33 pg/mL; 95% CI, −3.33 to −1.34; *p* < 0.001) and TNF-α (WMD, −1.61 pg/mL; 95% CI, −2.72, −0.51; *p* < 0.001), but not IL-6 and IL-8 levels, as a result of curcumin/turmeric supplementation [136].

CUR attenuates obesity-associated inflammation by inhibiting the activation of NF-κB, a key proinflammatory transcription factor. Its downregulation reduces the expression of molecules such as TNF-α, MCP-1, and IL-1, thus limiting the infiltration of macrophages into adipose tissue (Figure 3) [134,137]. These findings provide evidence for the anti-inflammatory effects of CUR supplementation and support further studies to confirm the dose, duration, and formulation to optimize its anti-inflammatory effects in obese humans with chronic inflammation.

### 4.3. Catechins

Green tea, made from the dried leaves of *Camellia sinensis*, is the most popular beverage. Compared to other teas (black or oolong), green tea contains the highest amount of catechin polyphenols, constituting about 35% of its total dry mass. Green tea prepared from a two-gram bag is estimated to contain about 500 mg of catechins. These are represented by (−)-epigallocatechin gallate (EGCG) (which accounts for 68-69% of catechins), (−)-epigallocatechin (EGC) (about 15–18% of catechins), (−)-epicatechin gallate (ECG) (about 5–6% of catechins), and (−)-epicatechin (EC) (about 2–5% of catechins) [138]. The main polyphenol in green tea, and thus the most studied, is EGCG. This bioactive compound from tea is characterized by various bioactivities, including anti-cancer, anti-diabetic, anti-proliferative, anti-obesity, antioxidant, and anti-inflammatory activities [56,139,140].

The anti-inflammatory potential of green tea catechins (GTCs) in obesity management has been shown in cell culture, animal, and human studies. In obese states, GTCs inhibit preadipocyte differentiation and decrease adipocyte proliferation. Moreover, they induce adipocyte apoptosis, suppress lipogenesis, and promote fatty acid oxidation [141]. It is worth adding that the potential mechanism by which EGCG acts as an antioxidant is the scavenging of reactive oxygen species, leading to the attenuation of NF-κB activity. It also controls other redox-sensitive transcription factors, such as Nrf2 and AP-1 [142]. As a result, GTCs decrease the concentrations of inflammatory biomarkers and OS in obese subjects (Figure 3) [81].

The flavan-3-ol (–)-epicatechin (EC) is another catechin widely present in the everyday human diet. Bettaieb et al. [117] showed that in palmitic acid-treated 3T3-L1 adipocytes, EC decreases TNF-α, IL-6, and MCP-1 concentrations and increases adiponectin levels. Moreover, mice on an HFD supplemented with EC had a lower expression of macrophage markers such as F4/80, TNF-α, and MCP-1 in VAT, as well as reduced NF-κB activity (Table 1). In another study conducted by Sano et al. [118], it was observed that in adipocytes co-cultured with LPS-induced macrophages, EC suppressed the gene expression of proinflammatory cytokines, such as IL-6, CCL19, Rantes, Ip-10, Saa3, Lbp, and Socs3. In the same study, C57BL/6J mice fed a normal or high-fat diet, with or without EC (20 mg/kg/day), showed decreased levels of TNF-α, IL-6, MCP-1, and Saa3 in white adipose tissue [118]. Similar results were obtained by Peng et al. [119]. In TNF-α-induced adipocytes, as well as in the WAT of HFD-fed mice, the authors observed the attenuation of adipose tissue inflammation and a reduction in adiposity in response to gallocatechin-(4->8)-gallocatechin-3-O-gallate (GC-(4->8)-GCG) exposure (Table 1). GTCs’ anti-inflammatory and anti-obesity effects have been shown in many other animal studies (Table 1) [120,143,144,145].

Several clinical studies have shown that green tea consumption affects obesity-induced inflammation in humans. Nonetheless, their results are inconclusive. Bogdański et al. observed that GTC consumption not only improves the metabolic profile (IR, lipid parameters, blood pressure) but also attenuates inflammatory states (by decreasing hs-CRP and TNF-α levels and increasing TAS) in patients with obesity-related hypertension (Table 1) [121]. Additionally, Bagheri et al. showed the positive influence of GTC (500 mg/d) supplementation together with endurance training on hs-CRP [146], as well as hs-CRP and IL-6 [147] values in overweight subjects. On the other hand, not all authors confirmed these results, indicating no effect of GTC intake on inflammatory markers [122,148]. Moreover, meta-analyses also provide ambiguous information. Rasaei et al. [149] analyzed sixteen RCTs, including 760 participants, and the results indicated that GTC supplementation had significant effects on TAC (WMD, 0.20 mmol/L; 95% CI 0.09 to 0.30, I2 = 98.6%, *p* < 0.001), which were associated with BMI and gender. No relationship between GTC supplementation and MDA has been observed, although a meta-regression analysis showed an inverse association between the dosage and MDA changes (r = −2117.18, *p* = 0.017). Other results obtained by Serban et al. [150] showed that GTC intake does not have a significant effect on plasma hs-CRP concentrations. Many more results were provided by Asbaghi et al. [151]. After analyzing eight articles with 614 T2DM patients, the authors found that GTC consumption significantly decreased CRP levels (WMD, −5.51 mg/dL, 95% CI −9.18 to −1.83, *p* = 0.003), with no effect on the plasma concentrations of TAC and MDA (0.02 mg/dL, CI, −0.06 to 0.10, and −0.14 mg/dL, CI, −0.40 to 0.12, respectively).

As mentioned above, there are a few different mechanisms thanks to which GTCs exhibit anti-inflammatory properties. The anti-adipogenic effect of GTCs, especially EGCG, occurs via the activation of AMPK, the main switch in energy metabolism regulation, as well as the attenuation of forkhead box protein O1 (FoxO1) and SREBP1c [152,153]. EGCG seems to increase the expression and phosphorylation of AMPK in adipocytes and the phosphorylation of acetyl CoA carboxylase (ACC), which results in a reduction in fatty acid esterification and, in turn, enhances their oxidation [154]. The anti-inflammatory effects of GTCs also involve their ability to modify the secretion of different adipokines. EGCG suppresses the secretion of resistin, a proinflammatory molecule, via ERK-dependent mechanisms. On the other hand, GTCs inhibit the expression of Krüppel-like factor 7 (KLF7) protein. KLF7 is a factor involved in reducing the expression and production of adiponectin and other adipogenesis-related genes, such as leptin, CCAAT/enhancer-binding protein α (C/EBPα), and PPAR-γ. Thus, its inhibition leads to the modification of the synthesis of the mentioned molecules [85,155]. It seems that further studies are needed, especially with the human population, to evaluate the exact mechanism of action of GTCs against obesity-induced inflammation.

## 5. Omega-3 Fatty Acids

Dietary fatty acids play an important role in humans as a main source of energy, elements of cell membranes, precursors of hormones, and immune complexes. Moreover, they protect organs from damage and participate in the absorption of fat-soluble vitamins [156]. Based on their structure, fatty acids can be divided into three categories, that is, saturated fatty acids (SFAs), monounsaturated fatty acids (MUFAs), and polyunsaturated fatty acids (PUFAs). PUFAs are classified into two subgroups (according to the position of the first double bond): omega-3 (n-3) and omega-6 (n-6) fatty acids. Omega-3 fatty acids have a carbon–carbon double bond situated three carbons from the methyl end of the chain. Health-promoting properties characterize PUFAs, and they have become valuable food constituents. Special attention is paid to the role of n-3 in attenuating inflammatory processes [157].

Omega-3 fatty acids are represented by α-linolenic acid (ALA), eicosapentaenoic acid (EPA), and docosahexaenoic acid (DHA). The main dietary sources of n-3 are marine fish (mackerel, sardines, mullet, salmon, tuna, trout, bluefish), nuts, and plant oils (rapeseed, linseed oil). While fishes are rich in long-chained EPA (C20:5) and DHA (C22:6), the plant sources of n-3 primarily deliver short-chained ALA (C18:3) [29]. The health benefits of ALA result from its ability to reduce the proinflammatory response, which has been proven in many studies [158,159,160]. These effects may be explained by the fact that, in mammals, ALA is converted to EPA and DHA during metabolic transformation. Thus, EPA and DHA seem to have stronger health protective effects due to their ability to incorporate into membrane lipids and to create precursors of anti-inflammatory lipid indicators, that is, novel specialized pro-resolving mediators (SPMs): resolvins, protectins, and maresins [29].

A large amount of evidence from both in vitro and in vivo studies indicates that n-3 significantly affects the different mechanisms responsible for the inflammatory response. In an in vitro co-culture model of murine 3T3-L1 adipocytes and RAW 264.7 macrophages, it was shown that DHA decreased the secretion of MCP1 and IL-6 from adipocytes and attenuated the mRNA expression of M1 polarization markers (*iNOS*, *TNF-α*, and *NF-κB*) while increasing the mRNA expression of IL-10, a solid anti-inflammatory cytokine (Table 2) [161].

Animal studies showed that EPA supplementation attenuates the inflammatory process by inhibiting cytokine expression (IL-6, TNF-a, MCP-1) in the stromal vascular fraction (SVF) as well as in AT from HFHS-fed mice. Moreover, supplementation with EPA suppresses CLS formation in mouse WAT and alters macrophage phenotypes to M2 (CD206) from M1 (CD11c) in the SVF by decreasing JNK and NF-κB activity [162]. In another study, it was shown that mice supplemented with long-chain n-3 PUFAs incorporated with phospholipids (n-3PL) or triacylglycerols (n-3TG) caused a reduction in proinflammatory processes and decreased the size of adipocytes (Table 2) [163]. It seems that n-3 supplementation prevents inflammation due to mechanisms involving enhanced PPAR-α signaling and diminished NF-κB activation [164].

Clinical studies have indicated that n-3 fatty acids attenuate obesity-associated chronic inflammation in adipose tissue. Itariu et al. showed that treatment with n-3 tended to decrease the expression of proinflammatory markers (such as IL-6) and increase the expression of anti-inflammatory molecules (such as adiponectin) in the subcutaneous adipose tissue (SAT) of severely obese nondiabetic patients (Table 2) [165]. In addition, other human studies showed an inverse association between EPA and DHA status and blood markers of inflammation, such as C-reactive protein [166,167,168] and cytokines [167,168], in obese subjects. On the other hand, some clinical studies could not confirm these results, indicating no effect of n-3 on inflammation [169,170].

It is worth mentioning that strong scientific evidence was derived by Schweitzer et al. [171] in their meta-analysis. After analyzing seven studies with 610 overweight and obese participants, the authors showed that n-3 long-chain polyunsaturated fatty acid intake promoted an overall reduction in serum proinflammatory eicosanoids and decreased the arachidonic acid COX-derived eicosanoid levels. In another umbrella meta-analysis, it was found that supplementation with n-3 PUFAs in adults reduced the concentration of CRP (effect size—ES = −0.40; 95% CI: −0.56 to 0.24, *p* < 0.001; I2 = 89.5%, *p* < 0.001), TNF-α (ES = −0.23; 95% CI: −0.37 to −0.08, *p* = 0.002; I2 = 60.1%, *p* < 0.001), and IL-6 (ES = −0.22; 95% CI: −0.39 to −0.05, *p* = 0.010; I2 = 66.2%, *p* < 0.001) under various health conditions [172]. These results were not confirmed in the case of ALA [173].

Omega-3 fatty acids impart anti-inflammatory activity to adipose tissue through a few pathways (Figure 3). One of them involves attenuating the proinflammatory transcription factor NF-κB by inhibiting the phosphorylation of its inhibitory subunit, that is, IκB [174]. Another involves the activation of the PPAR-γ receptor and a plasma membrane G protein-coupled receptor (GPR120), as well as the inhibition of arachidonic acid-mediated increases in proinflammatory eicosanoids by n-3. These eicosanoids act as ligands for GPR120. Thus, DHA activation of GPR120 reduces NF-κB activity in macrophages [175]. As mentioned above, n-3 fatty acids are recognized as precursors for the synthesis of SPMs (resolvins, protectins, and maresins), which are key examples of inflammation resolution agonists. In the case of n-3 deficiency, the promotion of various diseases with proinflammatory responses is initiated [176]. To sum up, the anti-inflammatory effects of n-3 include the inhibition of the secretion of proinflammatory mediators and a reduction in macrophage migration into AT. Moreover, it has been proven that n-3 intake prevents adipocyte proliferation, inhibits lipogenesis, and increases fatty acid oxidation, which may be considered their indirect anti-inflammatory effects [177].

**Table 2:** Omega-3 fatty acids and their effect on inflammation in obesity (results from selected in vitro, animal, and human studies).

Bioactive Compound	Experimental Model	Results	References
**Omega-3 fatty acids**	Co-culture model of murine 3T3-L1 adipocytes and RAW 264.7 macrophages incubated with 125 μM albumin-complexed DHA, EPA, palmitic acid (PA), or albumin alone (control) for 12 h	DHA: ↓ IL-6, MCP-1 ↓ mRNA of *Mcp1, iNOS, TNF-α,* and *NF-κB* ↑ mRNA of *Tgfb1* and *IL-10*	Boer et al. [161]
	Human adipose tissue (n = 8) and primary adipocyte cultures treated with endotoxin-free BSA conjugated with SFAs (lauric acid (LA) and palmitic acid (PA)) and PUFAs (EPA, DHA, and oleic acid (OA)) in a dose of 5 or 10 μM with or without LPS for 6 and 12 h	PUFA: ↓ TNF-α, MCP-1, IL-6	Murumalla et al. [178]
	SVF and adipose tissue from male HFHS-fed C57BL/6J mice on a diet supplemented with EPA (5% *wt*/*wt*) for 24 weeks 3T3-L1 adipocytes exposed to 250 μM palmitate for 30, 60, or 24 h with or without a 6 h pretreatment with 50 μM EPA-Na	EPA: ↓ IL-6, ↓ TNF-a, ↓ MCP-1, ↓ CLSs, ↓ CD11c, ↑ CD206 ↓ NF-κB, ↓ JNK	Yamada et al. [162]
	Four groups of C57BL/6 mice were fed LFD or HFD for 8 weeks. Two more groups of HFD were supplemented with n-3 PUFAs incorporated in the form of either phospholipids (n-3PL) or triacylglycerols (n-3TG)	PUFAs: ↓ MCP-1, IL-6, leptin, and 4-HNE, ↓ expression of *MCP-1* and *IL-6* in AT, and ↓ adipocyte size n-3PL: ↑ tocopherols in AT	Awada et al. [163]
	Severely obese nondiabetic patients (n = 55) scheduled to undergo elective bariatric surgery, taking 3.36 g n-3/d (EPA, DHA, n = 27) or an equivalent amount of butterfat as control (n = 28) for 8 weeks Randomized controlled trial	DHA and EPA: ↓ IL-6, ↓ TG, and ↓ expression of *MCP-1, CCl-3, IL-6*, and *CD40* ↑ Expression of *ADIPOQ* (adiponectin) in SAT	Itariu et al. [165]
	Overweight/obese pregnant women (n = 49) randomly assigned to receive DHA plus EPA (2 g/day) or the equivalent of a placebo twice a day from week 10–16 to term A randomized, double-masked controlled trial	DHA and EPA: ↓ hs-CRP ↓ TLR4, ↓ IL6, IL8, and TNF-α expression in maternal AT and placenta	Haghiac et al. [167]

Abbreviations: 4-HNE, 4-hydroxy-2-nonenal; AT, adipose tissue; BSA, bovine serum albumin; CCl-3, C-C Motif Chemokine Ligand 3; CLSs, crown-like structures; DHA, docosahexaenoic acid; EPA, eicosapentaenoic acid; IL-1, interleukin 1; IL-6, interleukin 6; IL-8; interleukin 8; IL-10, interleukin 10; JNK, Jun NH2-terminal kinase; hs-CRP, high-sensitivity C-reactive protein; LFD, low-fat diet; MCP-1, monocyte chemoattractant protein-1; NF-κB, nuclear factor kappa B; PGE2, prostaglandin E2; PUFAs, polyunsaturated fatty acids; SAT, subcutaneous adipose tissue; SFAs, saturated fatty acids; sICAM-1, soluble intercellular adhesion molecule 1; sVCAM-1, soluble vascular cell adhesion molecule 1; TG, triglycerides; TNF-α, tumor necrosis factor-α; TLR, toll-like receptor; WAT, white adipose tissue; VAT, viscera adipose tissue; ↑, increase; ↓, decrease.

## 6. Probiotics

In the multifactorial pathogenesis of obesity, much attention is paid to the gut microbiota (GM) and its influence on host metabolism [179]. The GM is defined as a complex and dynamic ecosystem of microorganisms inhabiting the gastrointestinal tract, composed of bacteria, fungi, archaea, viruses, and their genomes. The human GM consists of five main bacterial genera, including Firmicutes and Bacteroidetes, which account for about 90% of the total number of bacteria, and Proteobacteria, Actinobacteria, and Verrucomicrobia. Among Firmicutes, the human microbiota is mainly composed of butyrate-producing Eubacterium, Faecalibacterium, and Roseburia, as well as Lactobacillus, Ruminococcus, and Clostridium. Among the Bacteroidetes, there are Bacteroides, Prevotella, and Xylanibacter. In healthy adults, the most prevalent are Eubacterium, Clostridium, Ruminococcus, Lactobacillus, and Bacteroides [180]. However, the GM’s composition, diversity, and abundance may vary from person to person depending on many factors, including prenatal factors, age, ethnicity, the environment, medications and supplements taken, and overall lifestyle [180]. Apart from the inter-individual variation, three main GM enterotypes are distinguished depending on the dominant type of microorganisms in the environment: Bacteroides, Prevotella, and Ruminoccocus [181]. The GM is involved in various biological processes, including physiology and/or pathophysiology. It is a key regulator of the host’s energy homeostasis, the growth of pathogens, gut epithelial integrity, and immune function [180,182,183].

Previous studies have shown that GM dysbiosis, characterized by an increased Firmicutes-to-Bacteroideses ratio, reduced diversity, and changes in the activity of the GM, is closely linked to a variety of health problems, such as obesity and metabolic syndrome, cardiovascular diseases, and gastrointestinal disorders, as well as a chronic inflammatory disease [184,185]. Therefore, maintaining or restoring the balance of the GM through probiotics seems to be a promising and safe tool in obesity and obesity-related inflammation management.

The World Health Organization defines probiotics as live microorganisms that, when administered in adequate amounts, confer a health benefit to the host [186]. Available scientific studies have proven that interventions based on probiotic supplementation lead to beneficial changes in body weight and body composition, especially reductions in body fat, BMI, and waist circumference [187,188]. Moreover, by remodeling the GM, they are able to improve the cardio-metabolic profile [189]. Probiotics have been proposed as a new promising strategy in obesity treatment, not only because they are substances affecting body weight reduction or the restoration of glucose and lipid homeostasis but also because they positively affect markers of inflammation.

Of all the studies concerning probiotic intake, most have focused on Lactobacillus spp. In a study by Wu et al. [190], *L. fermentum* CQPC07 caused significant positive dose-dependent changes in several inflammatory cytokine levels and enhanced the antioxidative capacity. In another study, the antioxidant and anti-inflammatory properties of *L. fermentum* NCDC 400 in HFD-fed obese mice were described [191]. Similarly, a beneficial effect on obesity-induced low-grade inflammation was observed during *L. fermentum* CEC75716 supplementation in HFD-fed mice [192]. The positive influence on OS, inflammatory cell levels, and the expression of several inflammation-related genes has also been proven, among others, for *L. acidophilus* [193], *L. plantarum* 23-1 [194], *L. plantarum* ATG-K2 and ATG-K6 [195], *L. plantarum* KFY04 [196], *L. plantarum* OLL2712 [197], *L. plantarum* K50 [198], and heat-killed *L. plantarum* L-137 [199] in mouse model studies. In animal and clinical studies, metabolic parameter and inflammatory status improvements have been described during *L. reuteri* [200,201], *L. rhamnosus* LRH05 [202], *L. rhamnosus* LS-8 and *L. crustorum* MN047 [203], *L. gasseri* SBT2055 [204], *Latilactobacillus sakei* WIKIM31 [205], and *L. brevis* OPK-3 [206] administration. Several animal model studies also provided evidence that different strains of Bifidobacterium attenuate obesity-induced inflammation and OS [207,208,209]. Similarly, *BB. lactis* HN019 intake in patients with metabolic syndrome led to reduced IL-6 and TNF-α levels [210]. The anti-inflammatory properties of other bacterial strains, e.g., *Pediococcus pentococcus* PR-1, *Brevibacillus laterosporus* BL1, and *Saccharomyces boulardii*, have also been described in previous studies [211,212,213].

*Akkermansia muciniphila* is one of the most promising postbiotics in obesity and metabolic disorder treatment [214]. Moreover, its anti-inflammatory properties have also been described. In the study by Wu et al. [215], the colonization of *A. muciniphila* in a mouse model of immune-mediated liver injury reduced circulating LPS and significantly decreased the levels of proinflammatory cytokines.

In a study by Ashrafian et al. [216], the administration of *A. muciniphila* and its extracellular vesicles (EVs) positively influenced the intestinal barrier integrity, inflammatory state, fatty acid oxidation, energy homeostasis, and biochemical parameters (glucose and lipid levels) in mice with HFD-induced obesity [216], where the effect of *A. muciniphila*-derived EVs was greater compared with the bacterium itself. Similarly, in another study, *A. muciniphila* alleviated weight gain and reduced chronic low-grade inflammation in mice fed a normal chow diet [217]. After five weeks of supplementation, a decrease in the plasma levels of lipopolysaccharide (LPS)-binding protein (LBP) and leptin and the inactivation of LPS/LBP downstream signaling (mediated via decreased JNK phosphorylation and increased expression of IKBA) were described.

In the only clinical study conducted in this field, it was confirmed that *A. muciniphila* supplementation, apart from a significant effect on weight reduction and improvements in metabolic indicators, has an inflammation-modulating effect [218]. A 3-month supply of pasteurized *A. muciniphila* decreased LPS levels, enzyme DPP-IV activity, sCD40L levels, and the expression of the chemokine GRO. The positive metabolic effect can be explained by the effect of *A. muciniphila* on PPAR-α activation by mono-palmitoyl-glycerol [218].

Interventions using multi-strain probiotics, both in animal model studies and in clinical trials, also revealed the promising role of probiotics in enhancing obesity-induced inflammation and OS.

In an animal model study by Wang et al. [219], VSL#3 supplementation prevented weight gain and improved metabolic outcomes in mice with HFD-induced obesity. Additionally, VSL#3 effectively reduced adipose inflammation by restoring visceral adipose iNKT and stimulating iNKT cells to shift from a pre-inflammatory to an anti-inflammatory (IL-4+ iNKT cells) phenotype [219]. A similar immunomodulatory effect of probiotics on iNKT cells was described earlier by Ma et al. [208] in a mouse model of HFD-induced steatosis and insulin resistance. In a randomized, double-blind, placebo-controlled clinical study, the multi-strain probiotic Ecologic^®^ Barrier influenced TNF-α and IL-6 in a dose-dependent manner in postmenopausal women with obesity [220]. Similarly, a three-strain probiotic, including *L.salivarius*, *L. rhamnosus*, and *BB.animalis*, reduced TNF-α and beneficially modulated the proinflammatory adipokines leptin and adiponectin in children with excessive body mass [221]. The selected studies focused on evaluating the effects of probiotic therapy on obesity-induced inflammation are presented in Table 3.

Based on the above, probiotic administration seems to be a promising tool for treating obesity and improving obesity-induced chronic low-grade inflammation. As bioactive compounds with the ability to reverse the state of intestinal dysbiosis, probiotics have a positive effect on many different metabolic pathways, including glucose and lipid metabolism, energy homeostasis, antioxidant defense, and the modulation of the immune response via TLR4/NF-κB signaling pathway inhibition (Figure 3) [193]. Their administration reversed gut barrier dysfunction and, in consequence, led to an improvement in metabolic endotoxemia. Previous studies proved that probiotic intake decreased circulating LPS and LBP levels and attenuated local inflammation cascades by influencing nuclear factor-KB (NF-KB) and JNK and downregulating the expression of inflammatory cytokines such as TNF-α and IL-6, chemokines, adipokines, or intestinal inflammatory markers, e.g., zonulin or occludin [217].

## 7. Conclusions

Dietary patterns involving natural, bioactive food compound consumption seem to have a promising protective effect against obesity-induced inflammation, with limited harmful side effects. Numerous basic (in vivo and in vitro) as well as clinical studies have shown the relationship between the positive health outcomes of bioactive food compound intake and the attenuation of proinflammatory processes in the adipose tissue. They involve the modulation of the secretion of cytokines, adipokines, and hormones by adipocytes and their ability to regulate gene expression in adipose tissue. Although the exact mechanisms of bioactive food compounds’ action still need to be established, targeting the consumption and/or supplementation of food products with anti-inflammatory potential, such as polyphenols, omega-3 fatty acids, and probiotics, may represent a new approach for the prevention and treatment of obesity-induced inflammation, as well as its complications. Nonetheless, more clinical studies are warranted to establish the strategies for bioactive food compound intake. No less important is worldwide education about the advantages of bioactive food compound consumption, especially in the context of the prevention of Western-diet-induced obesity.

## Figures and Tables

**Table 3:** Supplementation with probiotics and their effects on inflammation in obesity.

Probiotics	Experimental Model	Results	References
*L. fermentum* CQPC07 (LF-CQPC07) in 2 doses: low dose (LD) of 1.0 × 10^8^ CFU/kg or high dose of (HD) −1.0 × 10^9^ CFU/kg)	6-wk-old C57BL/6 J mice (n = 60) on HFD supplemented with CQPC07 in LD or HD or placebo by 7 weeks	↓ IL-1β, TNF-α, IL-6, IFN-γ, ↑ IL-10, and IL-4 ↑ mRNA expression of CAT, GSH1, SOD1, SOD 2, and GSH-Px	Wu et al. [190]
*L. plantarum* L-137 (heat-killed; 0.002%)	Wk-old C57BL/6 J mice (n = 60) on HFD supplemented with *L. plantarum* L-137 or placebo	↓ Expression of inflammation-related genes (F4/80, CD11c, and IL-1β) in the epididymal adipose tissue ↓ LBP	Yoshitake et al. [199]
*L. reuteri* V3401 (5 × 10^9^ CFU/d)	Adults with MetS following healthy lifestyle recommendation (n = 53) supplemented with *L. reuteri* V3401 or placebo for 12 weeks Randomized, crossover, placebo-controlled, single-center trial	↓ IL-6 and sVCAM No significant effect on LPS, LBP, IL-8, CRP, TNF-α, MCP-1, or sICAM	Tenorio-Jiménez et al. [201]
*BB. breve* B-3 (5 × 10^10^ CFU/d)	Adult overweight volunteers (n = 52) receiving supplements with *B. breve* B-3 or placebo for 12 weeks Randomized, double-blind, placebo-controlled trial	↓ hs-CRP	Minami et al. [222]
VSL#3 VSL#3 was a high-concentration (1.5 × 10^9^ CFU/mouse/d) mixture of viable, lyophilized *Lactobacillus*, *Bifidobacterium*, and *Streptococcus thermophilus*	Wild-type (WT) (6-wk-old) male C57BL/6 mice (n = 16) and CD1d knockout (iNKT-cell-deficient) mice (n = 16) on an HFD or a normal-fat diet by 8 weeks CD1dKO (n = 16) and WT mice (n = 16) were then administered VSL#3 probiotics (active or heat—control group) by oral gavage for 4 weeks	VSL#3: ↓ adipose iNKT cell depletion and ↓ IL-6 and TNF- α Higher preventive effect against severe obesity development in wild-type mice than CD1dKO mice	Wang et al. [219]
Multi-species probiotic Ecologic^®^ Barrier containing *BB. bifidum* W23, *B. lactis* W51, *B. lactis* W52, *L. acidophilus* W37, *L. brevis* W63, *L. casei* W56, *L. salivarius* W24, *Lactococcus lactis* W19, and *Lactococcus lactis* W58 in 2 doses: LD (2.5 × 10^9^ CFU/d) or HD (1 × 10^10^ CFU/d)	Postmenopausal women with obesity (n = 81) receiving supplements with multi-species probiotic Ecologic^®^ Barrier in a low dose (LD) or a high dose (HD) or placebo for 12 weeks Randomized, double-blind, placebo-controlled clinical study	↓ IL-6 in both LD and HD groups ↓ TNF-α in HD group	Szulińska et al. [189]
Three-strain probiotic including *L. salivarius* AP-32 (109 CFU/d), *L. rhamnosus* bv-77 (109 CFU/d), and *BB. animalis* CP-9 (8 × 10^9^ CFU/d)	Overweight/obese children aged 6–18 years (n = 82) receiving supplements with a three-strain probiotic or placebo for 3 months Randomized, double-blind, placebo-controlled clinical study	↓ Leptin and TNF-α and ↑ adiponectin	Chen et al. [221]
*A. muciniphila* (109 CFU/200 μL) or extracellular vesicles (EVs) of *A. muciniphila* (10 μg protein/200 μL EVs)	8-wk-old male mice (n = 30) on HFD (group 1) or normal-fat diet (group 2) for 3 months After weight gain, both groups were divided into 3 subgroups receiving supplements with live *A. muciniphila* or EVs or placebo for 5 weeks	*A. muciniphila*: ↓ mRNA expression of TLR-4 and IL-6 genes in EAT, but no effect on TNF-α expression, ↑ expression of PPAR-α and PPAR-γ in adipose tissue, and ↓ TGF-β expression in EAT EVs: ↓ TNF-α, IL-6, and TLR-4 expression in HFD mice (greater than *A. muciniphila*), induced overexpression of PPAR-α in EAT in obese groups	Ashrafian et al. [216]
*A. muciniphila* (TCC BAA-835) 2 × 10^8^ CFUs/200 μL	6-wk-old male specific-pathogen-free (SPF)-grade C57BL/6 mice (n = 20) on normal chow diet supplemented with *A. muciniphila* or placebo for 5 weeks	↓ Plasma levels of lipopolysaccharide (LPS)-binding protein (LBP) and leptin and inactivation of LPS/LBP downstream signaling (e.g., decreased phospho-JNK and increased IKBA expression) in liver and muscle ↑ Anti-inflammatory factors such as α-tocopherol and β-sitosterol	Zhao et al. [217]
*A. muciniphila* Muc^T^ 3 × 10^9^ CFU/day	4-5-wk-old C57BL/6 mice (n = 22) with acute liver injury administrated *A. muciniphila* Muc^T^ or placebo by oral gavage for 14 days	↓ Proinflammatory cytokines MIP-1a, MIP-1b, and KC ↓ IFN-γ, IL-2, IL-1β, and IL-12p40 No effect on TNF-α ↓ Chemokines: MCP-1	Wu et al. [215]
*A.muciniphila* (alive or pasteurized) (1010 bacteria per day)	Adults with overweight/obesity and metabolic disorders (n = 32) receiving supplements with live or pasteurized *A.muciniphila* or placebo for 3 months Randomized double-blind placebo-controlled proof-of-concept trial	Only pasteurized *A.muciniphila* led to significant results: ↓ LPS, ↓ DPP-IV activity, and ↓ sCD40L levels and chemokine GRO No significant effect but a trend toward ↓ CRP and MCP-1	Depommier et al. [218]

Abbreviations: CAT, catalase; CPT1, carnitine palmitoyltransferase 1; CFU, colony-forming units; CRP, C-reactive protein; DPP-IV, dipeptidyl peptidase-IV; WT, wild type; IL, interleukin; GSH1, gamma glutamylcysteine synthetase 1; GSH-Px, glutathione peroxidase; GRO, growth-regulated oncogene/CXCL1; HFD, high-fat diet; iNKT, invariant natural killer T; interleukin(IL)-2; IFN-γ, interferon-γ; JNK, Jun NH2-terminal kinase; LBP, lipopolysaccharide binding protein; LPL, lipoprotein lipase; MCP-1, monocyte chemoattractant protein-1; MIP-1a; sCD40L, Soluble CD40 Ligand; TNF-α, tumor necrosis factor-α; SOD1, copper/zinc superoxide dismutase; SOD2, manganese superoxide dismutase; PPAR-α, peroxisome proliferator-activated receptor alpha; sICAM, soluble intracellular adhesion molecules; sVCAM-1, soluble vascular cell adhesion molecule 1, SPF, specific-pathogen-free; TGF-β, transforming growth factor β; WT, wild type; ↑, increase; **↓**, decrease.

## Data Availability

Not applicable.
